# Evaluation of formulated strigolactone analogs for Striga management in Kenyan agriculture

**DOI:** 10.1016/j.jafr.2025.101921

**Published:** 2025-06

**Authors:** Muhammad Jamil, Sylvia Mutinda, Jian You Wang, Damaris Barminga, Agnes Mwihaki, Lynet Navangi, Teresa O. Okiyo, Rohit H. Patil, Titus Ngatia, Patrick Mudavadi, Steven Runo, Salim Al-Babili

**Affiliations:** a The BioActives Lab, Biological and Environmental Science and Engineering, King Abdullah University of Science and Technology, Thuwal, 23955-6900, Saudi Arabia; b Department of Biochemistry, Microbiology and Biotechnology, Kenyatta University, 43844 - 00100, Nairobi, Kenya; c Kenya Agricultural and Livestock Research Organisation, Alupe Center, Busia, 399-50400, Kenya; d Kenya Agricultural and Livestock Research Organisation, Kibos Center, Kisumu, Kenya; e UPL House, Express Highway, Bandra-East, Mumbai, 400 051, Maharashtra, India; f UPL Kenya Leadership Team, 00100, Nairobi, Kenya; g Plant Science Program, Biological and Environmental Science and Engineering Division, King Abdullah University of Science and Technology (KAUST), Thuwal, 23955-6900, Saudi Arabia

**Keywords:** Field trials, Methyl Phenlactonoate, Nijmegen-1, Maize, Striga, Strigolactones

## Abstract

*Striga hermonthica*, an obligate root parasitic weed affecting cereal crops, poses a significant threat to global food security in Sub-Saharan Africa (SSA). Germination of Striga seed largely relies on signaling molecules released by the host roots, mainly strigolactones (SLs). Suicidal germination is an effective strategy for reducing Striga seed banks in infested soils by applying SL analogs in the absence of a host. However, the challenge remains in developing suitably formulated SL analogs for field application. In this report, we assessed the activity of two SL analogs, MP3 and Nijmegen-1, in both granular and liquid formulations in laboratory and greenhouse settings, and conducted mini-field and field trials to evaluate their effectiveness under farmers’ conditions, using maize as the host crop in Kenyan agriculture. We observed a significant induction of Striga seed germination reaching up to 56 % in laboratory germination bioassays and a reduction in Striga emergence by up to 77 % in greenhouse pot studies. In mini- and field trials in different infested fields, we recorded up to 80 % and 65 % reduction in Striga emergence, respectively. In conclusion, the formulated SL analogs demonstrate significant potential to reduce Striga infestation in maize fields in Kenya and are promising candidates for use by farmers due to their simplicity, ease of handling, stability, and effectiveness.

## Highlights

• Striga is an obligate root-parasitic weed that impacts cereal crops.
• Its seed germination is largely dependent on strigolactones released by host plants.
• Suicidal germination is a promising strategy for depleting Striga seed banks.
• A significant reduction in Striga infestation was observed with granular and liquid formulated MP3 and Nijmegen-1.
• The formulated SL analogs show strong potential to effectively reduce Striga infestations in maize fields in Kenya.

## Graphical abstract

**Figure:**
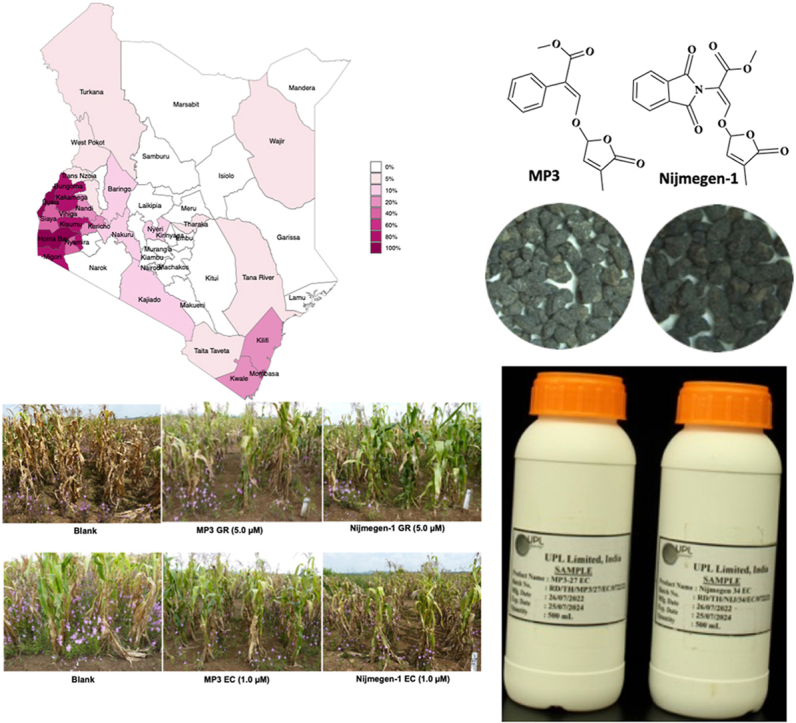

## 1 Introduction

Maize is an important commercial and staple food crop for millions of people worldwide [1]. It ranks among the top three cereal crops, alongside rice and wheat, providing essential human food, animal feed, industrial products, and biofuels [2]. In Africa, maize is a crucial source of livelihood and food security, supporting over 70 % of the population and contributing more than 25 % of their daily caloric intake [3,4]. Maize is cultivated on 42 million hectares in Sub-Saharan Africa (SSA), with a total production of 97 million tons [5]. However, the average maize yield in African regions is 2.5 tons per hectare (t/ha), which is approximately 62 % lower than the global average of 6.5 t/ha [5]. This low production level is attributed to various constraints, including low soil fertility, poor mechanization, and challenges from pests, diseases, and weeds. Among these, the hemi-parasitic plant *Striga hermonthica* (refer as Striga) poses a significant threat, severely impacting the livelihoods of smallholders in SSA [[6], [7], [8]].

**Fig. 1:**
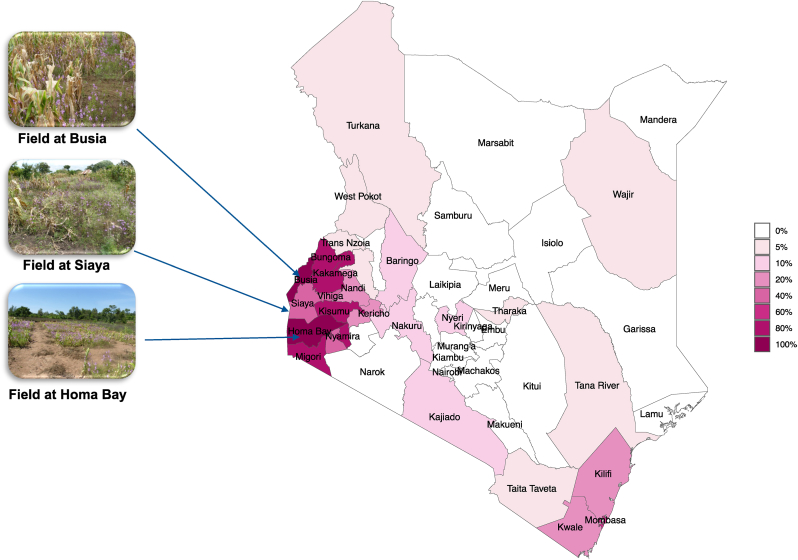
Overview of Striga infestation in Kenya. (A) Map of Striga infested areas in various parts of Kenya. The dark color showed the area with high levels of Striga infestation (>80 %). The medium color showed the area with moderate Striga infestation (50–60 %) and light color showed slight infestation levels (<20 %). (B) View of highly Striga infested maize farmer field in Busia, Siaya and Homabay, in Kenya selected for field trials.

In Kenya, maize is a staple food for over 90 % of the population [9], grown on 2.0 million hectares with a total production of 3.3 million tons [5]. Although the potential yield of maize in Kenya can reach up to 5.0 t/ha, the average yield is only 1.7 t/ha, which is significantly lower (74 % less) than the global average, and even below the African average of 2.5 t/ha [10]. One of the primary reasons for this low maize production is caused by Striga infection [11,12]. Striga infestation in the Lake Victoria Basin of Western Kenya has been documented as early as 1936 in farmers' fields [13]. Striga has now infested approximately 200,000 ha, covering 80 % farmland in Western Kenya (Fig. 1). This infestation has resulted in crop losses ranging from 35 % to 72 %, with an estimated value of around US$53 million [14,15]. As a result of Striga infestation, grain yields in the Lake Victoria Basin only range from 0.5 to 1.0 t/ha, which is less than 70 % of the potential yield [16].

The extensive seed production (50,000–200,000 per plant), seed longevity exceeding 15 years, widespread accumulation of seed banks over time, and a complex life cycle are significant obstacles to achieving sustainable Striga control [17]. Despite efforts by farmers and various organizations, utilizing both traditional and conventional Striga management strategies, the weed continues to pose a substantial challenge [18]. Numerous approaches, including traditional, chemical, biological methods, and host resistance, have been employed to combat Striga [19,20], yet none have successfully eradicated Striga. However, integrating two or more control methods has proven effective in reducing Striga infection but does not really reduce the Striga seedbank in the soil [21]. Hence, the few successfully survival parasites will mature and shed more seeds into the soil building up the seed density. Reducing Striga seed reservoirs in infested soil could be one of the most effective strategies to minimize the infestation levels and damage caused by this parasite [[22], [23], [24]].

Striga primarily relies on its host plant for water, nutrients, and photosynthates [25,26]. To facilitate this dependency, Striga has developed a host detection mechanism by recognizing host-derived signaling molecules known as strigolactones (SLs) [[27], [28], [29]]. The seeds of Striga germinate near host roots upon sensing host released SLs [30]. This reliance can be exploited through the application of synthetic SL analogs in the absence of a host—a technique referred to as "suicidal germination" [24,31]. This strategy has gained attention over the past three decades, leading to the development and screening of various germination stimulants for inducing suicidal germination in Striga seeds [[32], [33], [34], [35]]. Recently, based on their effectiveness, we have proposed two potent and straightforward SL analogs Methyl Phenlactonoate3 (MP3) and Nijmegen-1 to be used as suicidal germination agents, formulated in both liquid (EC-formulation) and granular (GRA) forms for practical field application [23,36]. However, detailed information on the efficacy, application methods, timing, and frequency of these suicidal agents is essential to develop a comprehensive Striga control strategy.

Herein, we evaluated the effectiveness of selected potent SL analogs against Striga infection in maize fields in Kenya. Specifically, we assessed and compared the efficacy of two SL analogs, MP3 and Nijmegen-1 [37], in both EC and granular formulations to explore the potential and future application of suicidal germination technology in Kenyan agriculture. For this purpose, we performed studies in laboratory, greenhouse and mini-boxes, and conducted field trials in Siaya, Homa Bay and Busia, the three Striga-infested regions in Kenya (Fig. 1).

## 2 Materials and methods

### 2.1 Plant materials and chemicals

**Fig. 2:**
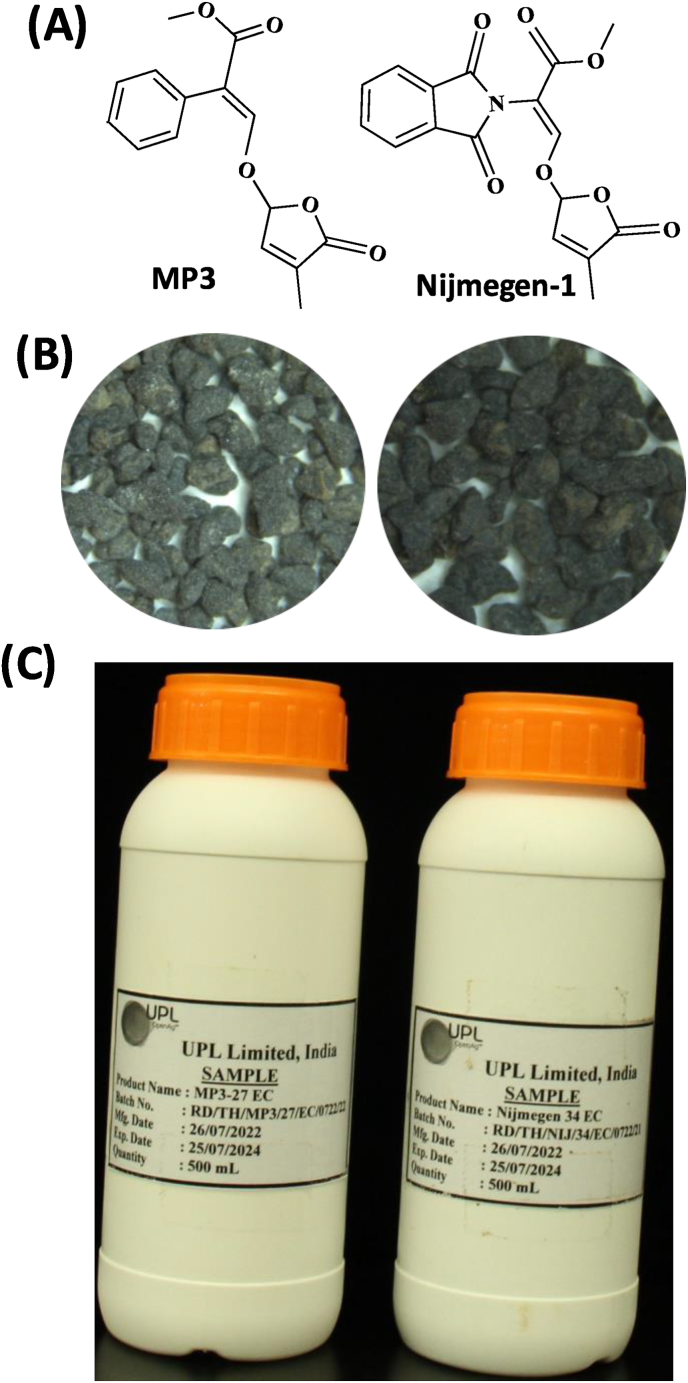
Structure of two selected SL analogs MP3 and Nijmegen-1 in granular and liquid formulation prepared by United Phosphorus Limited, India. (A) Structure of MP3 and Nijmegen-1, two potent germination stimulants, selected for formulation and field application. (B) View of granular formulation of MP3 and Nijmegen-1. (C) View of liquid formulation of MP3 and Nijmegen-1. The granular and liquid formulations of the two germination stimulants (MP3 and Nijmegen-1) were developed by United Phosphorus Limited (UPL), India. For the granular formulation, both analogs were coated onto commercially available blank roasted bentonite granules (7.5 % or 75 mg a.i./g). The required amount of active ingredient from MP3 or Nijmegen-1 was dissolved in solvent(s), followed by the addition of surfactant(s) and homogenized to form an Emulsifiable Concentrate (EC). The carrier granules were loaded into mixing equipment, where the mixture was evenly sprayed onto the granules. These granules were then coated with a solution containing the appropriate solvent and coating agent, followed by drying. For the liquid formulation, the required quantity of active ingredient from MP3 27 EC (27 mg/mL) or Nijmegen 34 EC (34 mg/mL) was dissolved in surfactant(s) and homogenized to produce an Emulsifiable Concentrate (EC).

The granular and liquid formulation of the two germination stimulants (MP3 and Nijmegen-1) were prepared by UPL, India (Fig. 2). For Lab. bioassays study, *Striga hermonthica* seeds were collected from a sorghum (*Sorghum bicolor*) field during 2020 in Sudan (provided by Prof. A. G. Babiker). Seeds of maize variety ‘30G18 - ZBAD’ were obtained from local market in Kenya.

### 2.2 Striga seed germination bioassays

The granular and liquid formulation of MP3 and Nijmegen-1 were first tested under lab conditions following the method described by Ref. [38]. Firstly, clean Striga seeds were surface sterilized with 50 % commercial bleach for 5 min and washed with sterilized milliQ water for six times to remove the bleach. The dry Striga seeds (∼50–100) were spread uniformly on a glass fiber filter paper disc (9 mm) in a laminar flow cabinet. Then 12 discs with Striga seeds were transferred in a plastic Petri plate containing one sterilized Whatman filter paper moistened with 3.0 mL sterilized milliQ water. The Petri plates were sealed with parafilm and were put in an oven at 30 °C for pre-conditioning. After 10 days, the discs with Striga seeds were dried in a laminar flow cabinet and six discs were put in a new Petri plate. A sterilized Whatman filter paper ring, moistened with 1.0 mL sterilized milliQ water was added in the plate to maintain internal moisture. The granular (5.0 μM) and liquid (1.0 μM) formulation of germination stimulants were applied (60 μL) on the top of each disc. The plates were sealed with parafilm and incubated at 30 °C in the dark for 24 h. The discs were scanned under a binocular microscope and germinated, and total seeds were counted by SeedQuant [39] and germination rate (in %) was calculated.

### 2.3 Striga emergence in pots under greenhouse conditions

The biological activity of the both formulation of the selected germination stimulants was tested in pots under greenhouse conditions as previously described [36]. A mixture of sand and soil (Stender, Basissubstrat) in 1:3 ratio was prepared and about 0.5 L of blank soil was added in the bottom of a 3.0 L perforated plastic pot. About 8000 Striga seeds (20 mg) were mixed uniformly in 1.5 L soil mixture and added on the top of the pot. After pre-conditioning phase for 10 days at 30 °C with light moisture, each pot was treated with liquid formulation (at 1.0 μM) and granular formulation (at 5.0 μM) of MP3 and Nijmegen-1 for two times. Four weeks after the last application, about 5 days old maize seedlings (cv 30G18 – ZBAD) were planted in the middle of each pot. The maize plants were allowed to grow under normal growth condition (30 °C, 65 % RH). After 70 days of sowing, Striga emergence was recorded in each pot and compared with the blank treatment.

### 2.4 Striga emergence under mini-box conditions

The two formulations of MP3 and Nijmegen-1 were also evaluated in mini-boxes at Kenyatta University, Kenya. A 60 cm deep poly fiber box with 1.0 × 1.0 m^2^ dimension was prepared. Each box was filled with sandy loam soil up to 30 cm depth. The top 10 cm of miniboxes were filled with soil infested with Striga seeds. The granular and liquid formulated Nijmegen-1 were applied (at 5.0 or 1.0 μM) for two times and blank treatment was included for comparison. After two months of last application, the host crop (maize) was sown in each box and emerged Striga plants were counted at 70 days after sowing (DAS).

### 2.5 Striga emergence under field conditions

The granular and liquid formulation of MP3 and Nijmegen-1 was further evaluated under artificially and naturally infested farmer fields in Western part of Kenya. The field experiments were established at three different locations near Lake Victoria Basin, Kenya (Fig. 1). Two highly Striga-infested farmer fields located near Homa Bay (0.5350° S, 34.4531° E) and Siaya (0.0626° N, 34.2878° E). Similarly, one artificially infested fields were established at KALRO, Busia (0.4611° N, 34.1102° E). In each field trial, the plots (4 × 4 m^2^) with various treatments were laid out by following randomized complete block design (RCBD) with five replications. All of the plots were spaced with 3 m distance to avoid any contamination of the treatments. Using a rainfed based protocol, we applied the germination stimulants on the onset of rainfall without any specified date. Each treated plot was supplied with required dose of granular and liquid formulation of strigolactone analogs with the assumption that the forthcoming rainfalls (∼20 mm) would dilute them to final concentration of 5.0 or 1.0 μM, respectively. For this purpose, laboratory spray bottles were used to apply SL analogs uniformly throughout the plot. We also included a treatment with the blank EC and blank granular as control treatment. Plots were weeded twice at 15 and 30 days after sowing (DAS) with hand hoeing before Striga emergence. Then, weeds other than Striga plants were hand-pulled until crop harvest. The maize (local cultivar Pioneer) planting was done after two months of last application, and data on Striga emergence were collected at 100–110 DAS, corresponding to the period of maximum emergence of Striga plants in the plots. After final counting of Striga plants from each plot, we harvested them to measure the host biomass and plant height.

### 2.6 Statistical analysis

All the data were collected following standard procedure and analyzed statistically using statistical software package R (version 3.2.2). One-way analysis of variance (ANOVA) with Least Significant Difference (LSD) multiple range test were used for analyzing the effect of two formulations of the germination stimulants on Striga infestation.

## 3 Results

### 3.1 Striga seed germination in response to the EC and GR formulation of SL analogs under lab conditions

**Fig. 3:**
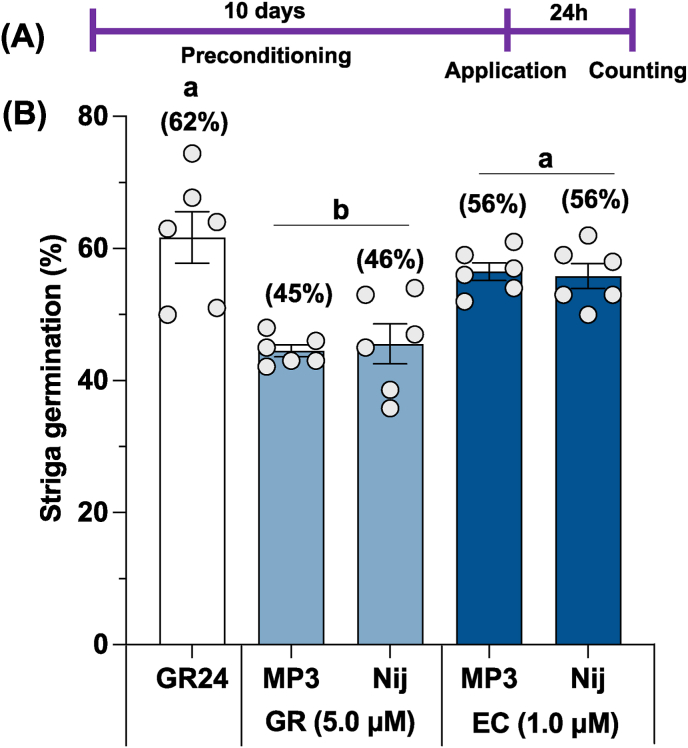
Striga seed germination activity of granular and liquid formulation of MP3 and Nijmegen-1. (A) Scheme of Striga germination bioassays study. (B) Striga seed germination in response to granular and liquid formulation of MP3 and Nijmegen-1. Values in parenthesis are showing percentage germination of Striga seeds (n = 6). For each germination stimulants, treatments with various letters differ significantly (p < 0.05).

The pre-conditioned Striga seeds were treated with two formulated SL analogs (MP3 and Nijmegen-1), and germination was observed after 24h treatment (Fig. 3A). We observed 62 % seed germination upon GR24, a canonical SL analog treatment; the EC formulation of the SL analogs MP3 and Nijmegen-1 showed about 45–56 % Striga germination, which was about 24 % higher than the GR formulation (46 %) (Fig. 3B). Interestingly, both the SL analogs exhibited similar activity with 56 % germination rate in EC formulation and 45 % in GR formulation.

### 3.2 Performance of the different formulations of SL analogs under greenhouse pot conditions

**Fig. 4:**
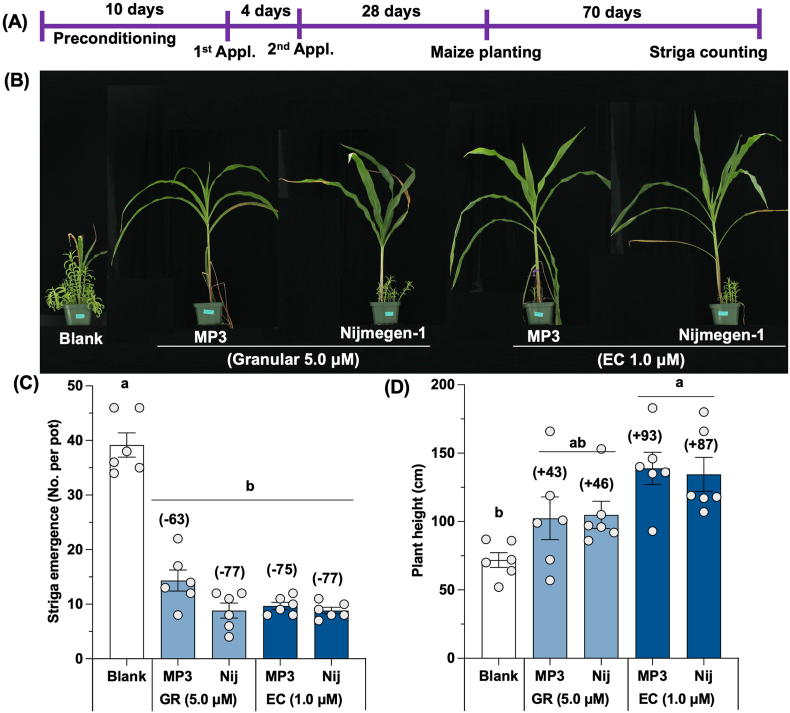
Effect of granular and liquid formulation of MP3 and Nijmegen-1 on Striga emergence in treated maize plant under greenhouse pot conditions. (A) Scheme of pot experiments. (B) View of Striga emergence in blank and treated pots. (C) Effect of granular and liquid formulation of the MP3 and Nijmegen-1 on Striga emergence and (D) maize plant height. The granular and liquid formulation of MP3 and Nijmegen-1 (at 1.0 or 5.0 μM) were applied two times in Striga infested pots to induce germination for four weeks. Striga emergence was counted 70 days after sowing of maize. Values of each bar show average number of Striga plants emerged in each pot (n = 6). For each germination stimulants, treatments with various letters differ significantly (p < 0.05). Values in parenthesis on the top of each bar are showing percentage increase (+) or decrease (−) of Striga emergence over blank treatment.

Next, both formulations of the two SL analogs were further evaluated under greenhouse conditions (Fig. 4A). Overall, the SL analogs in both EC and GR formulation statistically exhibited equal performance. The EC formulation of MP3 showed around 75 % decrease in Striga emergence, about 16 % higher than the reduction detected with the GR formulation of MP3 (63 %) (Fig. 4B and C). However, Nijmegen-1 showed similar activity with both EC and GR formulations, with a 77 % reduction in Striga emergence. As expected, the reduction in Striga emergence led to better growth of the maize host plant, indicated by an increase of 43–93 % in plant height of the maize host crop over blank treatment (Fig. 4D).

### 3.3 Comparison of the two formulations of SL analog under mini-field conditions in Kenyatta University

**Fig. 5:**
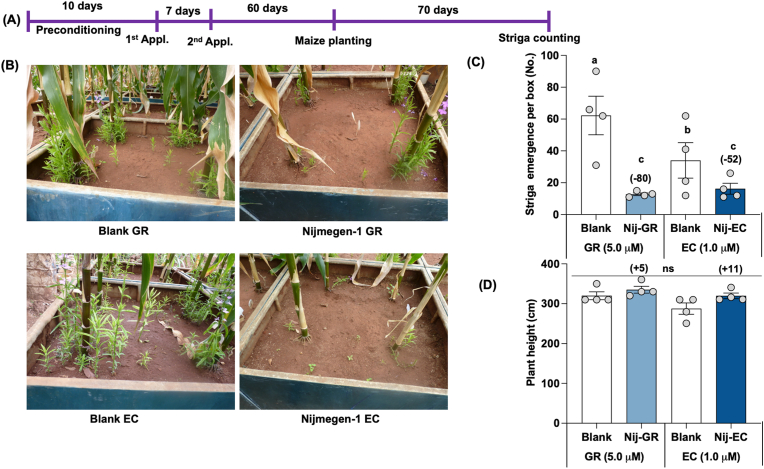
Testing the activity of granular and liquid formulation of Nijmegen-1 on Striga emergence under minibox conditions at Kenyatta University, Kenya. (A) Scheme of mini-box experiments. (B) View of Striga infested maize crop in miniboxes. (C) Striga emergence (D) Maize plant height per box in response to granular and liquid formulation of Nijmegen-1. Granular formulation of Nijmegen-1 (at 5.0 μM) and liquid formulation of Nijmegen-1 (at 1.0 μM) were applied in Striga infested mini boxes for two times. Striga emergence was counted at 90 days after maize planting. Values of each bar represent the average of Striga emergence (n = 4). The treatments with various letters differ significantly (p < 0.05). Values in parenthesis on the top of each bar are showing percentage increase (+) or decrease (−) of Striga emergence over blank treatment.

To explore the potential in real field conditions, we applied one selected SL analog Nijmegen-1 in both EC and GR formulation under mini-field conditions at Kenyatta University, Kenya (Fig. 5A). Nijmegen-1 in GR formulation showed an 80 % reduction in Striga emergence with respect to blank treatment, while its EC formulation exhibited a 52 % reduction in Striga emergence (Fig. 5B and C). We observed a 5 % increase in plant height in GR-Nijmegen-1 treated maize plants, while EC-Nijmegen-1 treated maize plants showed an 11 % increase in plant height compared to blank treatment.

### 3.4 Field assessment of the EC and GR formulation of SL analogs

**Fig. 6:**
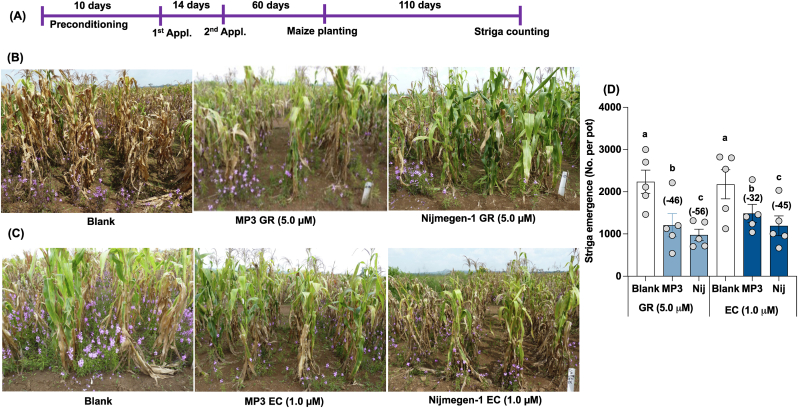
Effect of granular and liquid formulation of MP3 and Nijmegen-1 on Striga emergence in maize field in Busia, Kenya. (A) Scheme of field experiment. (B–C) View of maize field with Striga infestation in Busia, Kenya. (D) Striga emergence in maize field in response to various formulation of MP3 and Nijmegen-1. Various formulated SL analogs were applied for 2 times on the onset of rainfall. EC and granular formulated water (Blank) were used as control. Each bar represents average number of Striga emergence per plot (n = 5). For each SL analogs, treatments with various letters differ significantly (p < 0.05). Values in parenthesis on the top of each bar are showing percentage increase (+) or decrease (−) of Striga emergence over blank treatment.

Finally, we investigated the efficacy of both formulations of MP3 and Nijmegen-1 under farmer field conditions. We selected three highly infested areas around Lake Victoria Basin, Kenya. The first field trial was conducted with Kenya Agricultural and Livestock Research Organization (KARLO), Busia. Here, fields were artificially infested with Striga seeds. We applied the SL analogs to pre-conditioned Striga seeds two times and recorded Striga emergence after 110 days of application (Fig. 6).

In the Busia maize field, we observed a 46–56 % reduction in Striga emergence with the GR formulation of the two SL analogs compared to the blank treatment. Similarly, compared to blank treatment, we observed a 32–45 % reduction in Striga emergence with the EC formulation of the two SL analogs. The GR and EC formulation of Nijmegen-1 showed about a 45–56 % reduction in Striga emergence compared to blank. In comparison, the GR and EC formulation of MP3 showed about 32–46 % reduction in Striga emergence in comparison to blank (Fig. 6). The EC-formulated MP3 and Nijmegen-1 showed a 20–37 % increase in maize plant height over blank treatment, while the GR formulation of MP3 showed a 12 % increase in plant height (Supplementary Fig. S1A). The EC-formulated MP3 and Nijmegen-1 also showed a 73–165 % increase in maize total biomass over blank treatment, while the GR formulation of MP3 showed only an 18 % increase in total biomass. Although the grain yield in treated plots apparently showed more yield compared to untreated plots but high variation among replicated plots has made the effect non-significant.

**Fig. 7:**
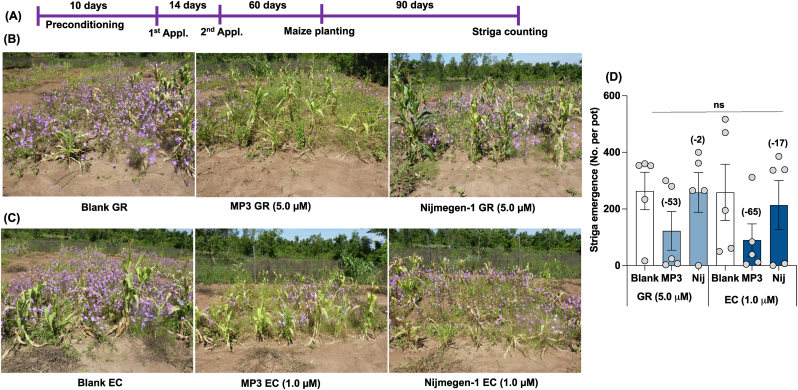
Effect of granular and liquid formulation of MP3 and Nijmegen-1 on Striga emergence in maize field in Homa Bay, Kenya. (A) Scheme of field experiment. (B–C) View of maize field with Striga infestation in Homa Bay, Kenya. (D) Striga emergence in maize field in response to various formulation of MP3 and Nijmegen-1. Various formulated SL analogs were applied for 2 times on the onset of rainfall. EC and granular formulated water (Blank) were used as control. Each bar represents average number of Striga emergence per plot (n = 5). For each germination stimulants, treatments with various letters differ significantly (p < 0.05). Values in parenthesis on the top of each bar are showing percentage increase (+) or decrease (−) of Striga emergence over blank treatment.

In the Homa Bay maize field, we observed a 2–53 % reduction in Striga emergence with the GR formulation of the two SL analogs compared to the blank treatment. Similarly, compared to blank treatment, we observed a 17–65 % reduction in Striga emergence with the EC formulation of the two SL analogs. The GR and EC formulation of Nijmegen-1 showed weaker activity (2-17) than MP3, which caused about 53–65 % reduction in Striga emergence (Fig. 7). The EC-formulated MP3 and Nijmegen-1 showed a 1–22 % increase in maize plant height over blank treatment, while the GR formulation of both SL analogs showed an 18–35 % increase in plant height (Supplementary Fig. S1B). We also observed an increase of 4–52 % in total maize biomass in GR-formulated SLs, while EC-formulated SLs showed a 10–66 % increase in total biomass production in maize fields. However, the high variation among replicated plots has made the effect of all treatments statistically non-significant.

**Fig. 8:**
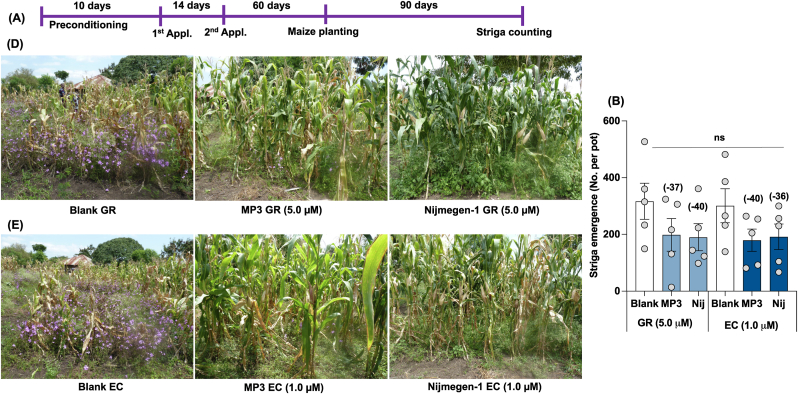
Effect of granular and liquid formulation of MP3 and Nijmegen-1 on Striga emergence in maize field in Siaya, Kenya. (A) Scheme of field experiment. (B–C) View of maize field with Striga infestation in Siaya, Kenya. (D) Striga emergence and plant height in maize field in response to various formulation of MP3 and Nijmegen-1. Various formulated SL analogs were applied for 2 times on the onset of rainfall. EC and granular formulated water (Blank) were used as control. Each bar represents average number of Striga emergence per plot (n = 5). For each SL analogs, treatments with various letters differ significantly (p < 0.05). Values in parenthesis on the top of each bar are showing percentage increase (+) or decrease (−) of Striga emergence over blank treatment.

Finally, we observed a 37–40 % reduction in Striga emergence with the GR formulation of the two SL analogs compared to the blank treatment in the Siaya maize field. Compared to blank treatment, we recorded a 36–40 % reduction in Striga emergence with the EC formulation of the two SL analogs. Both SL analogs' GR and EC formulations exhibited statically similar activity with a 36–40 % reduction in Striga emergence compared to blank treatment (Fig. 8). The EC-formulated MP3 and Nijmegen-1 showed a 2–25 % increase in maize plant height over blank treatment, while the GR formulation of both SL analogs showed a 2–15 % increase in plant height (Supplementary Fig. S1C). However, the high variation among replicated plots has made the effect on plant height by all treatments statistically non-significant.

## 4 Discussions

Reducing the seed bank in infested soils is a crucial strategy, achievable through the application of suicidal germination agents or formulated SL analogs. The effectiveness and formulation of these agents are critical for recommending them for practical field use [24,40]. As a case study, we evaluated two promising SL analogs, 'MP3′ and 'Nijmegen-1,' in both liquid and granular formulations under Kenyan mini-field and field conditions using maize as the host crop. The Striga seed germination rate was 56 % with the liquid formulation and 46 % with the granular formulation, indicating their bioactivity in lab conditions, nearly equivalent to the standard GR24 (62 %). The liquid formulation demonstrated approximately 24 % higher germination rate than the granular formulation due to its faster release than the GR formulation (Fig. 3). Indeed, these findings are consistent with previous studies [23,36]. Before transitioning to real-field applications, these compounds were first assessed under greenhouse-controlled conditions in pots containing Striga-infested soils with maize as the host crop (Fig. 4). Formulated MP3 and Nijmegen-1 were applied to each pot to induce suicidal germination, followed by the planting of a susceptible maize host to observe Striga emergence after 10 weeks of planting. A reduction of 63–77 % in Striga emergence achieved by both formulated germination stimulants under pot conditions demonstrates the potential of these suicidal agents for field application (Fig. 4). This significant reduction in Striga emergence is attributed to the effective induction of Striga seed germination by the suicidal agents, which easily reached the Striga seeds under controlled conditions. The reduction in Striga infection also led to an increase in maize plant height by 43–93 % compared to the blank treatment. These results are in consistent with previous studies [23,36]. Notably, neither formulation negatively impacted the host crop, further indicating their scope for field use.

Subsequently, we conducted a study under mini-field conditions at Kenyatta University. The mini-field, constructed from poly-fiber, measured 1 × 1 m^2^ with a depth of 60 cm. It was filled with blank soil to a depth of 30 cm, with the top 10 cm infested with Striga seeds. One of the selected SL analogs Nijmegen-1 was applied in both EC and GR formulations twice, and Striga emergence was counted 70 days after planting maize as the host crop. We observed an 80 % reduction in Striga emergence with the GR-formulated Nijmegen-1, while the EC formulation showed a 52 % reduction (Fig. 5). Notably, the GR formulation achieved a 28 % higher reduction than the EC formulation. The slow-releasing nature of the GR-formulated Nijmegen-1 might continuously induce Striga seeds over time, leading to greater reductions in Striga emergence. The timing between the final application and the planting of the host crop is crucial for achieving optimal results [24,31]. In fact, we still lack information on the stability of these compounds in the soil after application, so planting the host crop too soon could result in higher Striga infection due to soil-applied and host-released germination stimulants. Delayed planting and the sowing of a false host or resistant varieties a few weeks after the final application could lead to more effective Striga management [41,42].

Finally, we selected three highly infested field sites in Kenya to assess the efficacy of formulated germination stimulants against Striga. Two of the investigated sites were naturally infested farmer fields (Siaya and Homa Bay), while the third site, located at the KALRO Busia Research Station, was artificially infested. At the Busia Research Station, untreated blank plots showed high Striga infection, whereas GR-formulated treated plots exhibited a 45–56 % reduction in Striga emergence, and EC-formulated plots showed a 32–45 % reduction (Fig. 6). In the Homa Bay farmer field, we observed a 37–40 % reduction in Striga emergence with the GR formulation of both stimulants and a 36–40 % reduction with the EC formulation of both stimulants (Fig. 7). However, significant variation in Striga infection among individual plots resulted in statistically non-significant effects. Similarly, in the Siaya farmer field, we observed a 53 % reduction in Striga emergence with MP3 in GR formulation and a 65 % reduction with MP3 in EC formulation (Fig. 8). The formulations of Nijmegen-1 showed less effectiveness compared to MP3. However, significant variation in Striga infection among individual plots led to statistically non-significant results.

Further studies are required to determine the optimal amount, frequency, duration, and application methods for germination stimulants. Key questions remain about the stability of these stimulants in the soil, their persistence, and any residual effects. The responses underlying both the host and Striga to germination stimulants are also critical points that need further investigation. Different Striga ecotypes may react differently to various germination stimulants. The pattern of Striga infestation in maize fields suggests that the efficacy of suicidal agents may vary depending on the field location [43,44]. For instance, Nijmegen-1 was more effective in the Busia field, while MP3 showed more significant activity in Homa Bay. It is important to note the significant variation in Striga infestation levels across different field sites. In the maize field in Busia, we observed around 2000 Striga plants, whereas, in the Homa Bay maize field, the maximum infestation level was about 250 Striga plants per plot. The density of Striga seeds in an infested field can impact the efficacy of suicidal agents, with higher seed densities potentially reducing their effectiveness [8,45]. Additionally, soil pH, texture, and structure could significantly influence the activity of suicidal agents [24,31]. High pH, the low water-holding capacity of sandy soil, and uneven fields might lead to reduced efficacy of germination stimulants in infested areas [24]. Therefore, to prevent the further addition of Striga seeds in the year of application, we recommend planting a false host or resistant variety after applying the suicidal agents. Ongoing efforts focus on further validating this proposed suicidal technology across various locations in Kenya and other African countries. We observed a decrease in Striga emergence across various field trials, specifically in terms of the number of Striga plants emerging. The key question, however, lies in obtaining quantitative data on the actual reduction of the Striga seedbank in the soil over time. Although we collected soil samples before and after treatment applications for post-harvest analysis to evaluate the remaining Striga seedbank. Unfortunately, due to a lack of established protocols, sufficient manpower, and expertise, we were unable to complete this task. Once these limitations are addressed, we plan to pursue this analysis in future studies using Striga infested Kenyan soils.

## 5 Conclusion

In summary, the overall findings of this study highlight the great potential of formulated germination stimulants to combat Striga in maize fields in Kenya. Both formulations demonstrated significant effects on Striga seed germination under laboratory and minibox conditions, and led to a considerable reduction in Striga emergence in Kenyan maize fields. These formulated suicidal agents are simple, convenient, and easy to use, while also being stable, effective, and potentially remaining active in the soil for extended periods.

## CRediT authorship contribution statement

**Muhammad Jamil:** Writing – review & editing, Writing – original draft, Validation, Methodology, Investigation, Formal analysis, Data curation. **Sylvia Mutinda:** Methodology, Investigation, Data curation. **Jian You Wang:** Writing – review & editing, Methodology, Investigation. **Damaris Barminga:** Methodology, Investigation, Data curation. **Agnes Mwihaki:** Methodology, Investigation, Data curation. **Lynet Navangi:** Methodology, Investigation, Data curation. **Teresa O. Okiyo:** Methodology, Investigation, Data curation. **Rohit H. Patil:** Resources, Methodology, Investigation. **Titus Ngatia:** Methodology, Investigation. **Patrick Mudavadi:** Supervision, Methodology, Investigation. **Steven Runo:** Supervision, Methodology, Investigation. **Salim Al-Babili:** Writing – review & editing, Writing – original draft, Validation, Supervision, Methodology, Investigation, Funding acquisition, Conceptualization.

## Declaration of competing interest

The authors declare the following financial interests/personal relationships which may be considered as potential competing interests:Salim Al-Babili reports financial support was provided by Bill & Melinda Gates Foundation. Salim Al-Babili has patent LIQUID COMPOSITION OF STRIGOLACTONE ANALOGUES issued to 202221004961;PCT/IB2023/050754;2022-160-03;2022-160-04;2022-160-05;2022-160-06;2022-160-07;2022-160-08;2022-160-09. If there are other authors, they declare that they have no known competing financial interests or personal relationships that could have appeared to influence the work reported in this paper.

## Data Availability

Data will be made available on request.
