# Supplementary figures and images for: Evaluation of formulated strigolactone analogs for Striga management in Kenyan agriculture
Source: J Agric Food Res. 2025 Jun;21:101921. doi: 10.1016/j.jafr.2025.101921 (PMC12144315; doi:10.1016/j.jafr.2025.101921)

## Slide 1
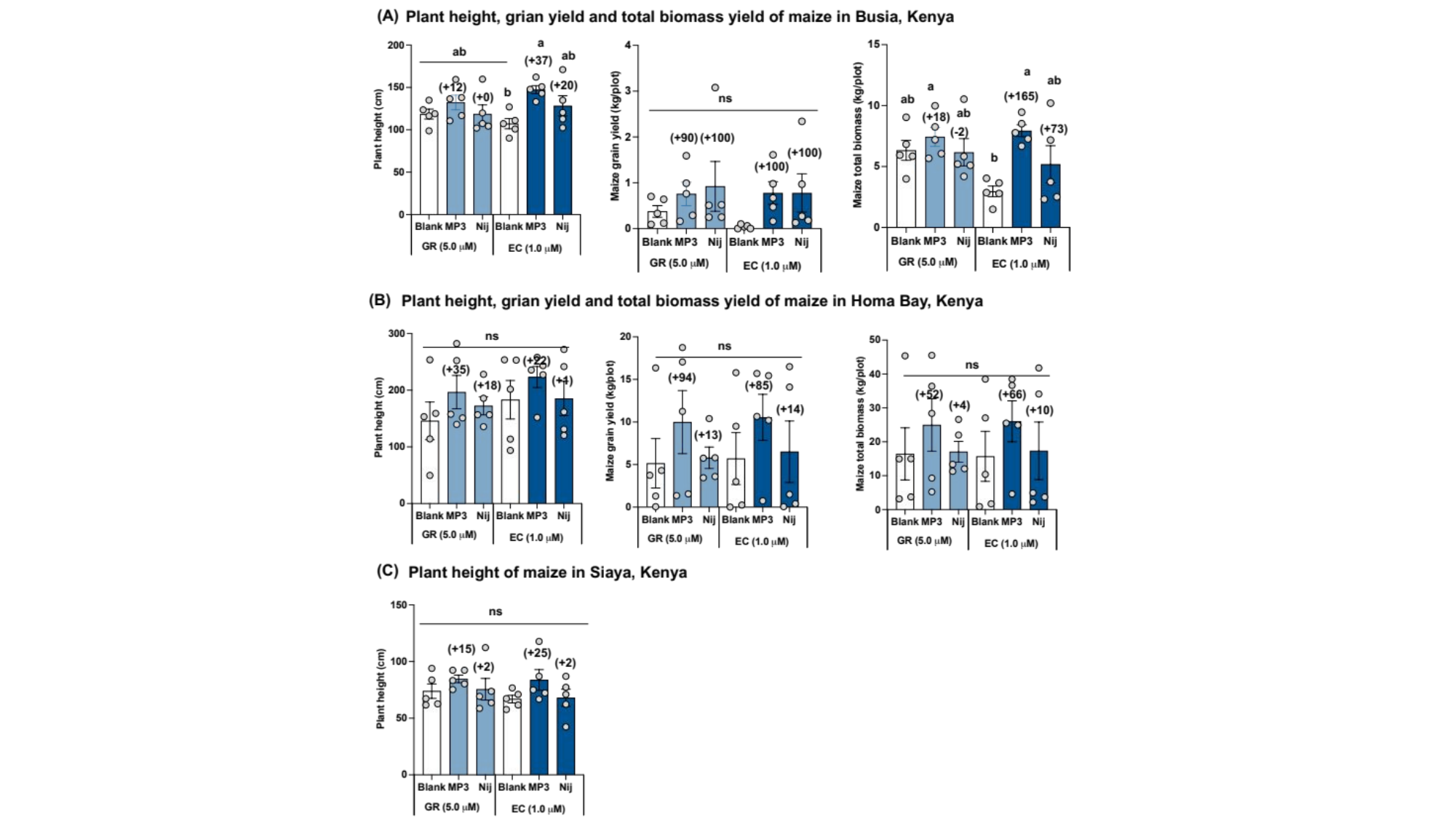

Supplement: Multimedia component 1 [file mmc1.pptx]
